# Supplementary material for: Flow-induced order–order transitions in amyloid fibril liquid crystalline tactoids
Source: Nat Commun. 2020 Oct 27;11:5416. doi: 10.1038/s41467-020-19213-x (PMC7591495; doi:10.1038/s41467-020-19213-x)
Supplement: Supplementary file 2 — Supplementary Information [file 41467_2020_19213_MOESM2_ESM.pdf]

Supplementary Information for:

Flow-induced order-order transitions in amyloid fibril liquid crystalline tactoids

Hamed Almohammadi[1], Massimo Bagnani[1], Raffaele Mezzenga[1,2*]

[1]Department of Health Sciences and Technology, ETH Zurich, Zurich, Switzerland.

[2]Department of Materials, ETH Zurich, Zurich, Switzerland.

*Correspondence to: raffaele.mezzenga@hest.ethz.ch

Supplementary Fig. 1

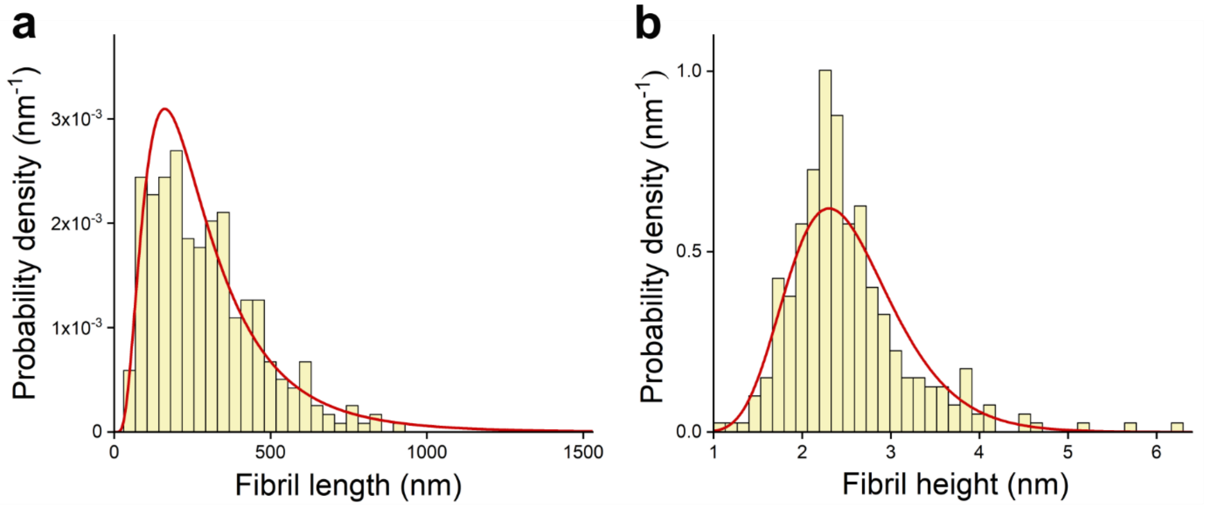

Supplementary Fig. 1 | Length and height distribution of amyloid fibrils. The solid curves are the lognormal distribution fitted to data where n = 300. **a** The arithmetic and weighted mean length values are $L_{f,m}$ = 303 nm and $L_{f,w}$ = 424 nm, respectively. The fitting parameters are: $\mu_{fitting}$ = 5.5 ± 0 and $\sigma_{fitting}$ = 0.6 ± 0. **b** The mean arithmetic fibrils diameter (height) value is $D_{f,m}$ = 2.5 nm with fitting parameters $\mu_{fitting}$ = 0.9 ± 0 and $\sigma_{fitting}$ = 0.3 ± 0.

Supplementary Fig. 2

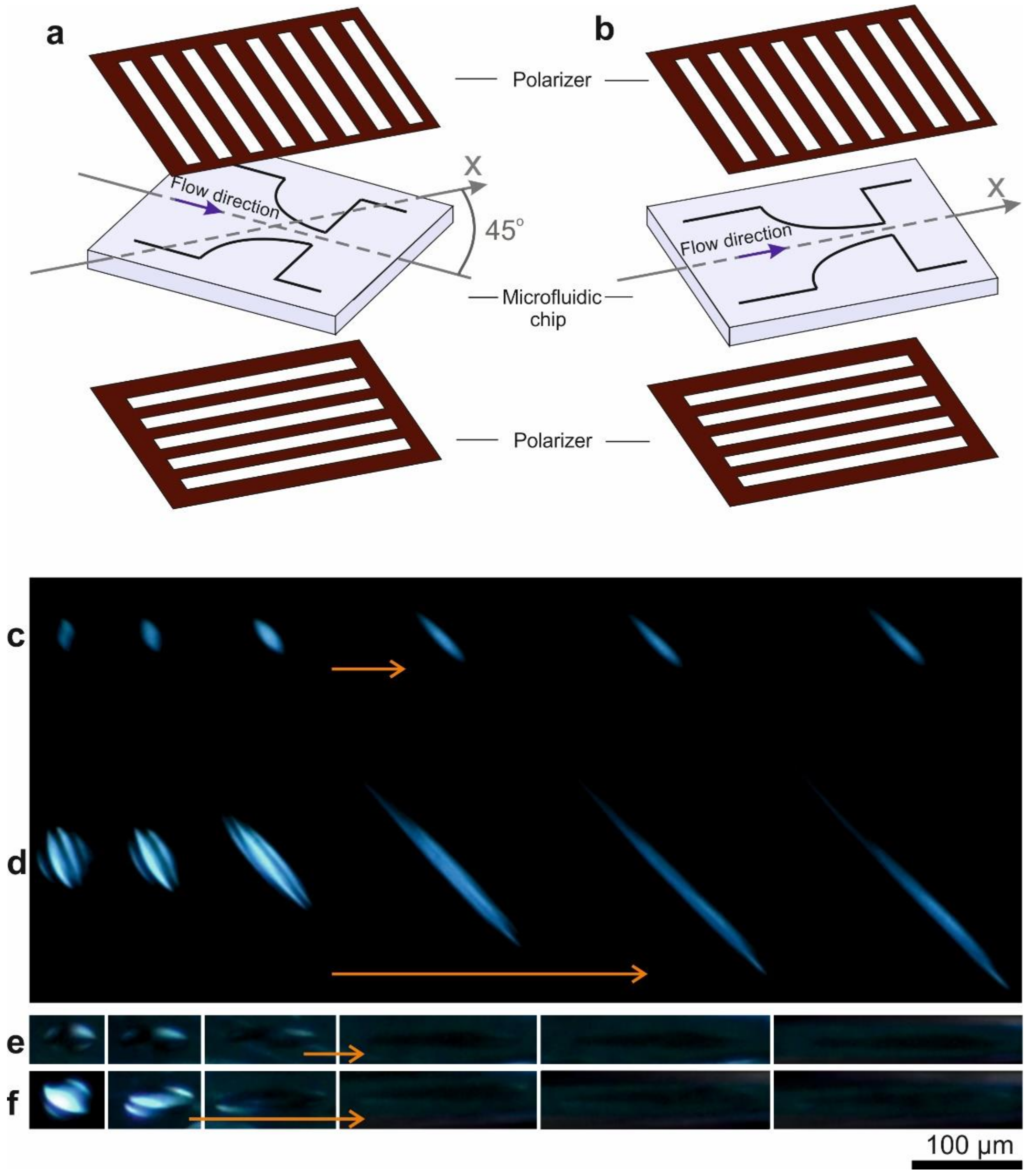

Supplementary Fig. 2 | Deformation-induced order-order transitions in tactoids. The transition is observed at two different angles with respect to fixed crossed polarizers. **a** The microfluidic chip is placed in a way to have 45º between the extension ($\dot{\varepsilon}_{xx}$) direction and crossed polarizers. **b** The microfluidic chip is rotated 45º degree compared to (**a**), resulting in 0º or 90º between extension direction and crossed polarizers. **c, d** The bipolar and cholesteric tactoids are elongated in configuration (**a**) resulting in observing of the elongation of the tactoids in 45º with respect to crossed polarizers. The phase transition from bipolar and cholesteric to homogenous is shown with red arrow (the extended red arrow in (**d**) shows the phase that is defined as transition phase). **e, f** The bipolar (**e**) and cholesteric (**f**) tactoids are elongated using configuration depicted in (**b**). The transition to homogenous is determined when the tactoids become invisible. Scale bar stands for panels **c-f**.

Supplementary Fig. 3

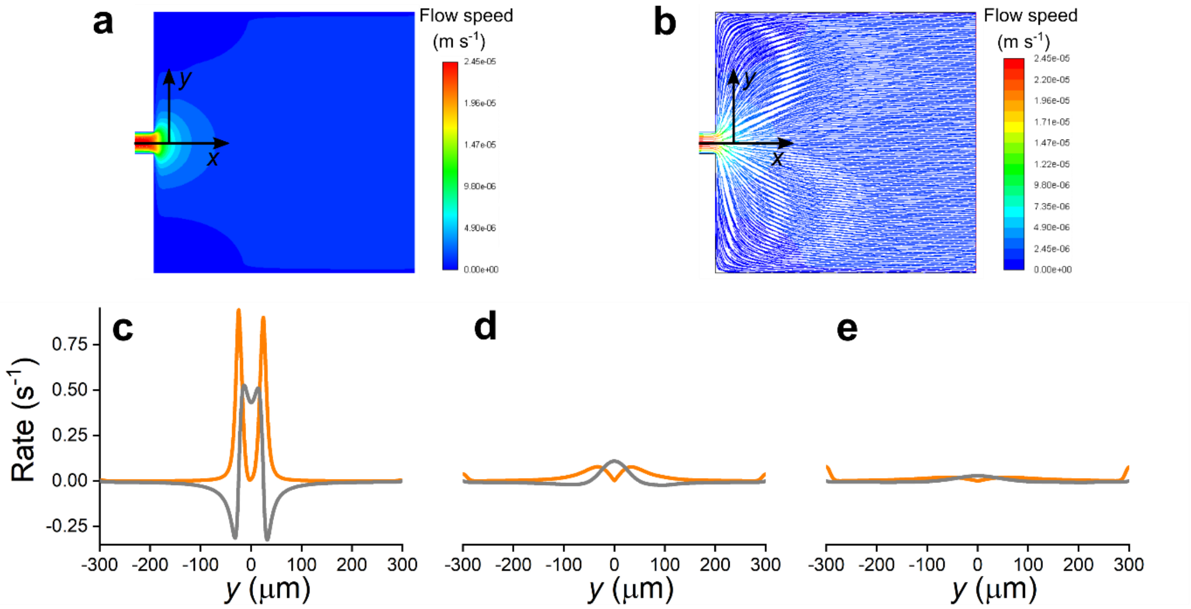

Supplementary Fig. 3 | Simulation results of the fluid flow in expansion section of the used microfluidic channel. a Velocity magnitude counter. **b** Streamlines colored by flow speed magnitude. **c, d, e** Comparison of $\dot{\varepsilon}_{yy}$ (gray line) and $|\dot{\Gamma}_{xy}|$ (orange line) at different distance from throat in the channel, where the distances from throat are 10 (**c**), 50 (**d**), and 100 (**e**) μm.

Supplementary Fig. 4

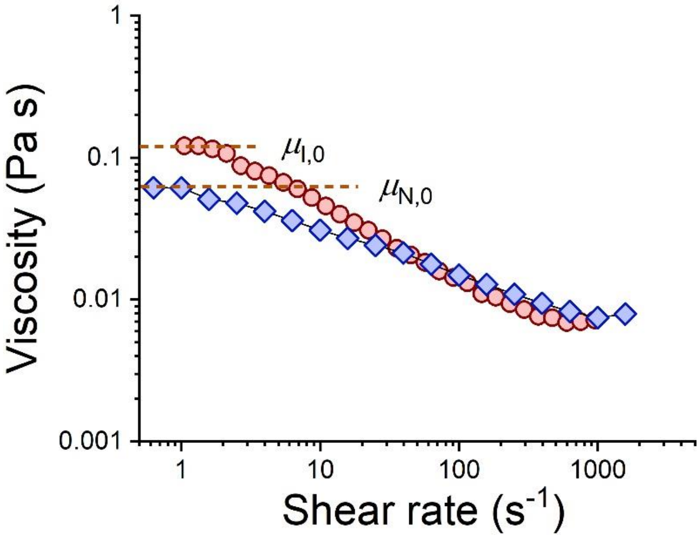

Supplementary Fig. 4 | Viscosity of the isotropic and nematic phases of amyloid fibrils versus shear rate. The dashed line denotes zero shear viscosity that is equal to 0.121 and 0.061 Pa s for isotropic and nematic phases, respectively.

Supplementary Fig. 5

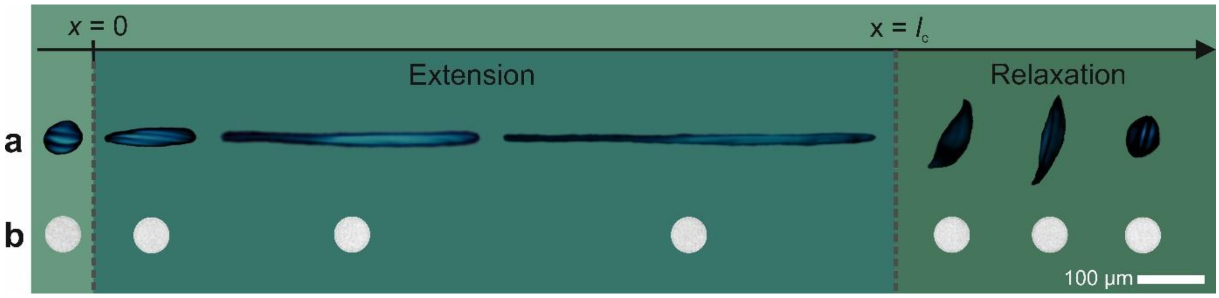

Supplementary Fig. 5 | Comparison of deformation of liquid crystalline tactoid and oil droplet. The extension rates are (**a**) 0.017 and (**b**) 0.020 s^{-1}. While the tactoid reaches to aspect ratio of approximately 38 or the final to initial length ratio of approximately 10 (**a**), the oil droplet remains almost unreformed (**b**).

Supplementary Fig. 6

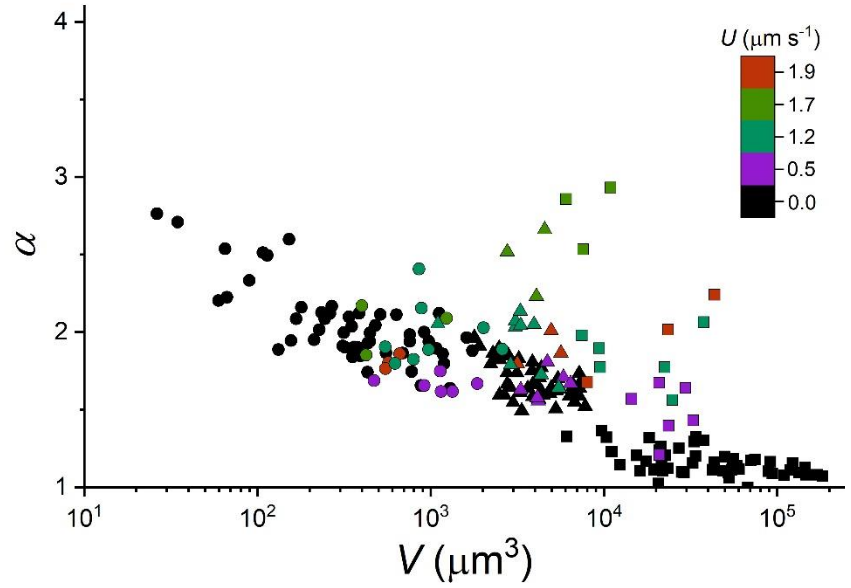

Supplementary Fig. 6 | Aspect ratio of the tactoids as a function of their volume. The tactoids are at equilibrium condition (U = 0 m s^{-1}) or under a flow field with velocity U in the microfluidic channel**.** The velocity (U) values are the flow speed that are tested in this study to get different extension rate in Fig. 3. The black symbols (U = 0 m s^{-1}) refer to the sample that is placed in a cuvette.

Supplementary Fig. 7

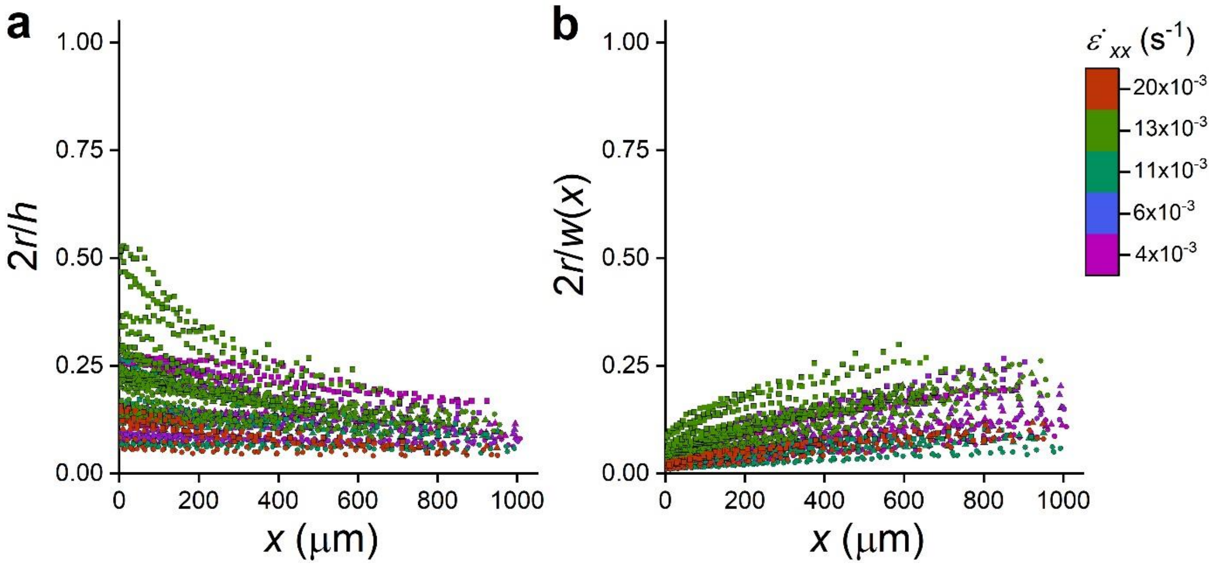

Supplementary Fig. 7 | Tactoids short axis evolution versus channel height and width. Evaluation of the $2r/h$ (**a**) and $2r/w(x)$ (**b**) for different classes of tactoids considered in this study at different extensional rate during deformation along the contraction zone.

Supplementary Fig. 8

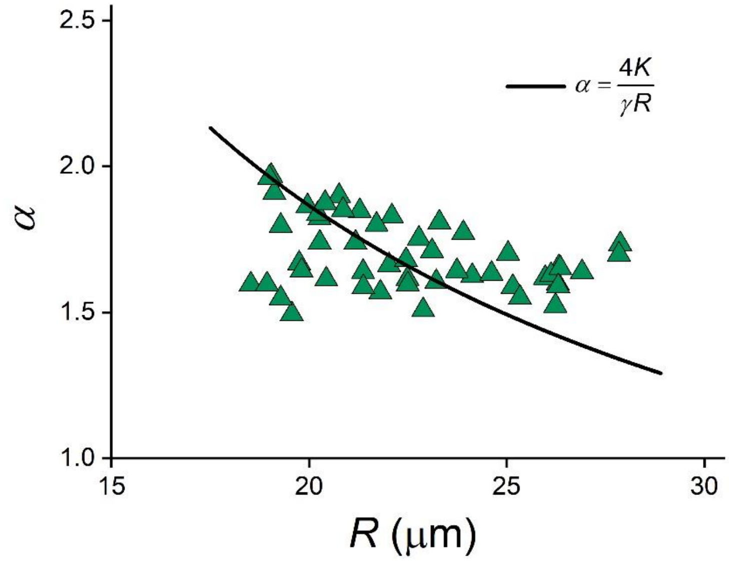

Supplementary Fig. 8 | Aspect ratio of the bipolar tactoids as a function of their long axis at equilibrium. The solid curve obeying $\alpha = \frac{4K}{\gamma R}$ indicates the best fit to data with fitting parameter $K = 1.0 \times 10^{-11}$ N.

Supplementary Fig. 9

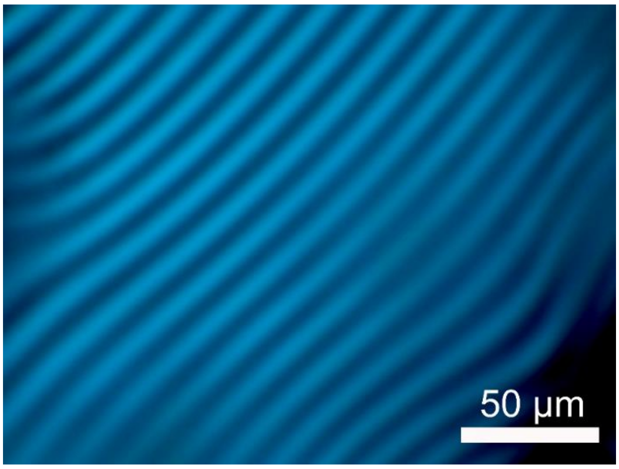

Supplementary Fig. 9 | Cholesteric bulk phase of amyloid fibrils solution. The value of P_∞ is found to be 25.6 µm.

Supplementary Note 1

Length and height distribution of amyloid fibrils

Supplementary Fig. 1 presents the distribution of length of fibrils L_f and height of the fibrils which corresponds to the fibrils diameter considering a cylindrical approximation D_f, obtained by analyzing the AFM images using the FiberApp software developed by Usov and Mezzenga (Supplementary Ref. 1). The lognormal distributions were fitted in MATLAB resulting in fitting parameters of $\mu_{\text{fitting}} = 5.5 \pm 0$ and $\sigma_{\text{fitting}} = 0.6 \pm 0$ for the length and $\mu_{\text{fitting}} = 0.9 \pm 0$ and $\sigma_{\text{fitting}} = 0.3 \pm 0$ for the height distributions.

Supplementary Note 2

Determination of order-order transition

Here we provide further details to support deformation-induced order-order transitions between different classes of liquid crystalline tactoids, as shown in Supplementary Fig. 2. In the main text (Fig. 2), we provided the tactoids deformation when their long axes (or short axis) is placed close to 45º relative to crossed polarizers during the deformation in contraction zone. Such configuration allows to have long and short axis of the tactoids to be visible for unambiguous measurement and study of deformation. To do so, we simply placed the microfluidic chip in a way that the flow direction is in approximately 45º with respect to crossed polarizers, as the tactoids long axis align in the flow direction in the contraction zone, Supplementary Fig. 2a. In this setup, the order-order transition of the bipolar to homogenous tactoids is identified when curvilinear orientation of the director in the bipolar that leads to appearance of the invisible corner for the tactoid changes to uniformly aligned director field that is seen as almost uniform white texture, Supplementary Fig. 2c. We determined the cholesteric to homogenous transition, when the stripped texture of the tactoids changes to uniform white texture for the tactoid, Supplementary Fig. 2d.

To further support the order-order transition from bipolar to homogenous tactoid, following the experimental protocol designed in Supplementary Ref. 2 to distinguish different classes of tactoids by rotating the tactoids under crossed polarizers, we performed experiments in such a way to have the tactoids to be parallel to one of the crossed polarizers. This is achieved by placing the microfluidic channel in such a fashion that the flow direction is parallel to one of the crossed polarizers,

Supplementary Fig. 2b. In this configuration, the bipolar tactoid is determined when a central dark cross is seen, while the homogenous tactoid should be invisible (Supplementary Ref. 2). Thus, using this setup, the transition from bipolar to homogenous tactoid should happen, when the texture with central dark cross becomes invisible. This is clearly seen in Supplementary Fig. 2e.

Note that the tactoids continuously elongates and the internal configuration of the tactoids adapts itself to the new configuration (Supplementary Fig. 2). During such elongation, as the curvature of the interface decreases, the internal configuration continuously transforms in the axial direction. This happens until a critical aspect ratio is reached, above which the tactoids cannot hold anymore the bipolar configuration and the transition to homogenous structure takes place. Thus, we can state that the whole process of the transition from bipolar tactoid at rest to homogenous is a continuous transition. However, from an experimental point of view, the tactoids configuration becomes well distinguishable as bipolar and homogenous, respectively below and above the critical aspect ratio, so that we can state that the transition happens in proximity of such a critical aspect ratio.

Supplementary Fig. 2f shows the cholesteric to homogenous tactoid transition. Upon elongation of the cholesteric tactoid whose long axis is almost parallel to one of the crossed polarizers (but not exact parallel, as we want to show its stripped texture) and reaching a critical elongation ratio, the stripped texture becomes invisible. This shows order-order transition of cholesteric to homogenous phases, and rules out the possibility of transition to any other class other than homogenous.

It should be noted that although in equilibrium condition, the order of the transition is from homogenous to bipolar and then to cholesteric with an increase in volume and decrease in aspect ratio, here due to flow-induced deformation, bipolar configuration is not observed when a cholesteric tactoid is elongated. To support this argument, we look at the transition from a theoretical perspective and compare the results with the data presented in Figure 4a in the main text. Let's assume that there is a cholesteric, to bipolar and then to homogenous tactoids transition. This implies that there should be a transition from bipolar to homogenous in the aspect ratio that is less than approximately 17 (see Fig. 4a), as we already see homogenous tactoids for the aspect ratio higher than approximately 17 when the cholesteric tactoids are deformed. However, following the theory (Equation 3 in the main text) that predicts bipolar to

homogenous tactoids transition, the expected aspect ratio for bipolar to homogenous transition for a tactoid with initial cholesteric configuration is approximately 56. This prediction is much higher than the range that we see homogenous tactoids and shows the invalidity of the assumption that there is a cholesteric, to bipolar and then to homogenous tactoids transition.

Note that, in Supplementary Fig 2e and f, after the transition, the homogenous tactoids with director field parallel to one of the crossed polarizers, look darker than the isotropic medium. This is so as statistically the number of the rods that are in parallel to one of the crossed polarizers is higher when one looks though the tactoids in the chip compared to isotropic medium. This is also reflected in Fig. 2 in Supplementary Ref. 2 when a homogenous tactoid is place in parallel to one of the crossed polarizers. In Supplementary Fig. 2c and d the inhomogeneous intensity of birefringence along the deformed tactoids is related to the heterogeneity of the thickness of the tactoids.

Supplementary Note 3

Tactoids rotation in expansion zone

To investigate the rotation of the tactoids, we performed a simulation using ANSYS Fluent software in identical flow speed and channel geometry to that of Figure 2, i.e. U = 1.5 µm s^{-1}, $w_u = w_d$ = 600 µm, w_t = 50 µm, and h = 100 µm (Supplementary Fig. 3). The simulation was performed for an isotropic non-Newtonian fluid having the same properties of the isotropic phase of our system. The results show that there is no vortex formation after fluid leaves the contraction part, Supplementary Fig. 3b. In addition, we are able to see how the velocity decreases from contraction part to downstream section, Supplementary Fig. 3a. Theoretically, the average flow speed should decrease by the inverse ratio of the channel width, i.e. $U/\mathrm{u}_t = w_t/w_d$ as volumetric flow rate Q is constant for a given flow condition, u_t shows the flow speed at the throat section of the contraction part. Thus, considering that in Fig. 2, the channel geometry is w_d = 600 µm and w_t = 50 µm, the average flow speed is expected to decrease by 1/12 as $U/u_t = w_t/w_d$ = 1/12.

We also monitored the extension rate of the flow in y direction in the downstream channel $\dot{\varepsilon}_{yy}=\partial u_y/\partial y$ and compared it to the shear rate in the flow direction $\dot{\Gamma}_{xy}=\partial u_x/\partial y$, see Supplementary Fig. 3c to e.

Essentially, as it is well explained in Supplementary Ref. 3, when the $\dot{\varepsilon}_{yy}$ is strong compared to $|\dot{\Gamma}_{xy}|$, (i.e. $\dot{\varepsilon}_{yy}/|\dot{\Gamma}_{xy}|>0.14$), cylindrical particles rotate by approximately 90º, aligning their long axes perpendicular to flow direction. Our results show that in the center of channel right after the contraction zone the extension rate $\dot{\varepsilon}_{yy}$ is higher than shear rate $|\dot{\Gamma}_{xy}|$, explaining the mechanism behind the rotation of the tactoids shown in Fig. 2. In fact, stronger extension rate $\dot{\varepsilon}_{yy}$ when compared to $|\dot{\Gamma}_{xy}|$ forces the tactoids to align their long axis in the direction of $\dot{\varepsilon}_{yy}$. It can also be seen that as we move away from the throat the values of $\dot{\varepsilon}_{yy}$ and $|\dot{\Gamma}_{xy}|$ get closer to each other, Supplementary Fig. 3c to e.

Supplementary Note 4

Determination of the Viscosities

For hydrodynamic analysis in this study, we assume the viscosity of the medium and tactoids to be equal to viscosity of the isotropic and nematic phases, respectively. Thus, viscosity of the isotropic and nematic phases are measured following the description provided in Methods. The desired amount of suspension from isotropic and nematic phases from a suspension of the amyloid fibrils that was already phase separated were taken and analyzed. As shown in Supplementary Fig. 4, the zero shear viscosity of the nematic phase is lower than the isotropic phase, which can be related to alignment of the fibrils in nemaic phase. Note that, although the viscosities of the liquid crystalline phases vary depending on the shear rate, following the common assumption in the context of the droplet deformation (Supplementary Ref. 4), we used zero shear viscosity values for hydrodynamic analysis in the main text.

Supplementary Note 5

Oil-in-water control experiments

To show the effects of the low interfacial tension of the tactoids, experiments with simple fluids (oil droplet in water-glycerol mixture), with interfacial tension of order of approximately 0.01 N m^{-1}, are performed and compared against the tactoid (Fig. 2f or Supplementary Fig. 5). To be systematic in the comparison, the relevant parameters in the droplet deformation (Supplementary Ref. 4), i.e. droplet size, the viscosity of the droplet, viscosity of the medium, and accordingly the viscosity ratio of droplet to medium, as well as extension rate, were kept essentially the same. We used water-glycerol mixture with viscosity of 0.122 Pa s (is taken from Supplementary Ref. 5) as medium phase and olive oil with

viscosity of 0.063 Pa s (measured directly) for droplet phase, having similar viscosity as liquid crystalline system where the viscosity of the surrounding phase and tactoid are, respectively, 0.121 Pa s and 0.061 Pa s (see Supplementary Note 4). The droplet size and extension rate of the olive oil droplet are chosen to be almost equal to the tactoid that is shown in Fig. 2c (or Supplementary Fig. 5a), i.e. olive oil: droplet radius = 27 µm and extension rate = 0.020 s^{-1}, tactoid: equivalent radius of tactoid = $(r^2R)^{1/3}$ = 26 µm and extension rate = 0.017 s^{-1}. Comparing Figures 2c and 2f (or Supplementary Fig. 5a and Supplementary Fig. 5b), it is clear that, while tactoid reaches to aspect ratio of approximately 38 (or the final to initial length ratio of approximately 10), the oil droplet remains almost undeformed. This clearly illustrates the remarkable effects of the very low interfacial tension of the tactoids.

Supplementary Note 6

The shape of the tactoids in the straight channel

As it is mentioned in the main text, the tactoids need to travel the straight channel with velocity U before entering the contraction zone. Here we compare the aspect ratio of the tactoids as a function of their volume at equilibrium condition (the sample is placed in a cuvette) and in the microfluidic channel before entering to the constriction zone at $x = 0$ in Fig. 1 (defined as initial shape in the main text), see Supplementary Fig. 6. The velocity values reported in Supplementary Fig. 6 are the flow speed values that are tested in this study to get different extension rate in Fig. 3. As it can be seen, the aspect ratios of homogenous tactoids in all tested conditions and bipolar tactoids at low flow velocity (U = 0.5 µm s^{-1}), are in the range of equilibrium state. The aspect ratio of cholesteric and bipolar tactoids are higher than the equilibrium state and around three at the highest flow speed. However, such deviation of the initial shape of the tactoids from equilibrium state when these are compared to the range of aspect ratio that the tactoids experience are very small (3/25 = 0.12). Thus, it enables to extend the linear deformation argument of the tactoids for this small deviation range as well and rationalize that Equation 1 (in the main text) can be used to predict the deformation of the tactoids from its equilibrium state.

Supplementary Note 7

Tactoids short axis evolution versus channel height and width

Supplementary Fig. 7a and b shows respectively the evaluation of the $2r/h$ and $2r/w(x)$ for all the different classes of tactoids at different extensional rate during deformation along the contraction zone. For the vast majority of the tactoids studied in this work, the $2r/h$ and $2r/w(x)$ values are less than 0.3 confirming that we are far from Hele-Shaw limit for droplet or $2r < h$ and $2r < w(x)$ where axisymmetric geometry for the droplet does no longer stand valid.

Supplementary Note 8

Determination of the density, interfacial tension, anchoring strength, splay and bending elastic constant, and twist elastic constant

Density. To get density of continues phase to be used in Reynolds number in the main text, similar to viscosity, we assume the density of the medium to be equal to density of the isotropic phase, ρ_I, and write it as a function of amyloid fibrils density, ρ_{amy}, and water density, ρ_{H2O}, using:

$$\rho_I = \frac{m_{amy}+m_{H_2O}}{V_{amy}+V_{H_2O}} = \frac{\rho_{amy}V_{amy}+\rho_{H_2O}V_{H_2O}}{V_{amy}+V_{H_2O}} \tag{1}$$

where m_{amy}/V_{amy} and m_{H2O}/V_{H2O} are the mass/volume of the amyloid fibrils and water, respectively. To be able to get ρ_I in Supplementary Equation 1, we relate V_{amy} and V_{H2O} using following relation to isotropic phase concentration, c_I :

$$c_I = \frac{m_{amy}}{m_{amy}+m_{H_2O}} = \frac{\rho_{amy}V_{amy}}{\rho_{amy}V_{amy}+\rho_{H_2O}V_{H_2O}} \tag{2}$$

Substituting c_I = 0.02 wt, ρ_{amy} = 1.3 g cm^{-3} (taken from Supplementary Ref. 6), and ρ_{H2O} = 1.0 g cm^{-3} in Supplementary Equation 2, we get $V_{amy} = 0.016\ V_{H2O}$. This together with density values substituted in Supplementary Equation 1 results in ρ_I = 1.005 g cm^{-3}.

Interfacial tension. The interfacial tension of the tactoids is estimated using universal scaling law that predicts the interfacial tension of the phase separated colloidal suspension of hard rods as (Supplementary References 7-8):

$$\gamma = b\frac{k_B T}{L_{particle}D_{particle}} \tag{3}$$

where k_B is Boltzmann constant, T is the temperature, $L_{particle}$ and $D_{particle}$ are the length and diameter of the hard rods, respectively, that are taken to be equal to $L_{f,w}$ and $D_{f,m}$ of the fibrils here. The term b is a constant that is taken to be 0.3 value (Supplementary References 2,8). Substituting all the parameters, we get interfacial tension to be $\gamma = 1.1 \times 10^{-6}$ N m^{-1}.

Anchoring strength. To get the anchoring strength, we followed the supplementary Ref. 9 showing linear dependence of anchoring strength on fibrils length. Thus, knowing the anchoring strength for fibrils with average length of 401 and 525 nm to be 1.45 and 1.8, respectively, from Supplementary Ref. 2 and Supplementary Ref. 9, we estimated the anchoring strength for fibrils with average length of 303 nm used in this work to be 1.17. Similar anchoring strength value, 1.06±0.27, is found using Wulff construction $\omega = (\alpha/2)^2$, that is for when anchoring strength is higher than one and α is the aspect ratio of the homogenous tactoids at equilibrium condition (Supplementary Ref. 9). This also confirms the linear dependence of the anchoring strength on fibrils length as discussed in Supplementary Ref. 9.

Splay and bending elastic constant, *K*. The splay and bending elastic constant (K) is found by fitting the relation $\alpha = \frac{4K}{\gamma R}$ to the data that shows the aspect ratio of bipolar tactoids as a function their long axis at equilibrium condition, Supplementary Fig. 8. This derivation that relates the shape of the bipolar tactoids to K is provided in Supplementary Ref. 2 by minimizing the energy of the bipolar tactoids at equilibrium condition.

Twist elastic constant, *K*₂. Similar to Supplementary Ref. 2, we find K_2 using (Supplementary Ref. 10):

$$K_2 \sim k_B T L_{particle}{}^2 n \tag{4}$$

where n is the number of rod-like objects. Having the all values for the parameters in Supplementary Equation 4 and K_2 to be 0.559×10^{-12} N for the amyloid fibrils with weighted mean length (L_{502}, lets use the length value as a subscript in this section) of 502 nm and concentration (c_{502}) of 0.013 g cm^{-3} from Supplementary Ref. 2. We take these values as a reference and find the K_2 for the amyloid fibrils suspension used in this study that is with weighted mean length (L_{424}) of 424 nm and concentration of c_{424} = 0.022 g cm^{-3}, using:

$$K_{2_{424.5}} = (\frac{T_{424}}{T_{502}})(\frac{L_{424}}{L_{502}})^2(\frac{n_{424}}{n_{502}})K_{2_{502}} \tag{5}$$

where n parameter can be substituted by $c/(\rho_{\text{particle}} V_{\text{particle}})$ with c the concentration, and ρ_{particle} and V_{particle} ($\sim D_{\text{particle}}{}^2 L_{\text{particle}}$) the density and volume of rod-like objects, respectively. To get the volume of the amyloid fibrils, one need to take the fibrils as a double stranded cylinder. This together with simplification of Supplementary Equation 5 can give:

$$K_{2_{424.5}} = \left(\frac{L_{424}}{L_{502}}\right)\left(\frac{c_{424}}{c_{502}}\right)\left(\frac{D_{502}}{D_{424}}\right)^2 K_{2_{502}} \tag{6}$$

Now substituting all of the above reported parameters and the values of D_{502} = 2 nm and D_{424} = 1.25 nm (these are diameter for protofilament, Supplementary Ref. 11), we get twist constant for our used amyloid fibrils suspension in this study to be $K_{2_{424}}$= 2.0 × 10^{-12} N.

Supplementary Note 9

Determination of P_∞

Supplementary Fig. 9 shows the bulk cholesteric phase of the used amyloid fibrils ($L_{\text{f,m}}$ = 302 nm), showing the P_∞ to be 25.6 μm. This, together with the P_∞ = 20 μm for fibrils with $L_{\text{f,m}}$ = 401 nm and P_∞ = 15 μm for fibrils with $L_{\text{f,m}}$ = 525 nm in, respectively, Supplementary Ref. 2 and Supplementary Ref. 9, further supports the argument in Supplementary Ref. 9 pointing at an increase in the pitch of the cholesteric phase with a decrease in fibrils length.

Supplementary References

1. Usov, I. & Mezzenga, R. FiberApp: an open-source software for tracking and analyzing polymers, filaments, biomacromolecules, and fibrous objects. *Macromolecules* **48**, 1269-1280 (2015).
2. Nyström, G., Arcari, M. & Mezzenga, R. Confinement-induced liquid crystalline transitions in amyloid fibril cholesteric tactoids. *Nat. Nanotech.* **13**, 330 (2018).
3. Trebbin, M. et al. Anisotropic particles align perpendicular to the flow direction in narrow microchannels. *Proc. Natl Acad. Sci. USA* **110**, 6706–6711 (2013).
4. Milliken, W. J., & Leal, L. G. Deformation and breakup of viscoelastic drops in planar extensional flows. *J. Non-Newton. Fluid Mech.* **40**, 355-379 (1991).
5. Cheng, N.-S. Formula for the viscosity of a glycerol─water mixture. *Ind. Eng. Chem. Res.* **47**, 3285–3288 (2008).
6. Nyström, G., Fong, W. K., & Mezzenga, R. Ice-templated and cross-linked amyloid fibril aerogel scaffolds for cell growth. *Biomacromolecules* **18**, 2858-2865 (2017).
7. van der Schoot, P. Remarks on the interfacial tension in colloidal systems. *J. Phys. Chem. B* **103,** 8804–8808 (1999).
8. Koch, D. L. & Harlen, O. G. Interfacial tension at the boundary between nematic and isotropic phases of a hard rod solution. *Macromolecules* **32**, 219–226 (1999).
9. Bagnani, M., Nyström, G., De Michele, C. & Mezzenga, R. Amyloid fibrils length controls shape and structure of nematic and cholesteric tactoids. *ACS Nano* **13**, 591-600 (2018).
10. Sato, T. & Teramoto, A. On the Frank elastic constants of lyotropic polymer liquid crystals. *Macromolecules* **29**, 4107–4114 (1996).
11. Adamcik, J. et al. Understanding amyloid aggregation by statistical analysis of atomic force microscopy images. *Nat. Nanotechnol.* **5,** 423–428 (2010).
